# Feeding-Based RNA Intereference of a Gap Gene Is Lethal to the Pea Aphid, *Acyrthosiphon pisum*

**DOI:** 10.1371/journal.pone.0048718

**Published:** 2012-11-07

**Authors:** Jianjun Mao, Fanrong Zeng

**Affiliations:** The Key Laboratory of Pest Management in Crops, Ministry of Agriculture, Institute of Plant Protection, Chinese Academy of Agricultural Sciences, Beijing, People's Republic of China; University of Massachusetts Medical, United States of America

## Abstract

The gap gene *hunchback* (*hb*) is a key regulator in the anteroposterior patterning of insects. Loss-of-function of *hb* resulted in segmentation defects in the next generation. In this paper, *hb* expression level was investigated at different developmental stages of the pea aphid, *Acyrthosiphon pisum* (*Ap*). *Aphb* mRNA was most early detected at the first instar stage and showed an incontinuous increase in the whole life cycle. Ingested RNA interference was performed at the second instar stage to knockdown the *Aphb* expression. Continuous feeding of *Aphb* double-stranded RNA mixed in artificial diet led to reduction of *Aphb* transcripts and rise of insect lethality. These results indicated that *hunchback* was a good RNAi target in the management of insect pests.

## Introduction

Aphids, which are considered as one of the main animal pests in agriculture, feed exclusively on plant phloem sap by inserting the needle-shaped mouthparts into sieve elements. Many of 5,000 aphid species attack crops and ornamental plants, and cause great losses worldwide both by direct feeding and by vectoring various plant viruses [1], [2]. Compared with insects with chewing mouthparts, aphids are more difficult to control because pesticide sprayed on plant surface almost can not be absorbed via digestive tract of sap-sucking insect pests.

RNA interference (RNAi) is the sequence-specific gene silencing induced by double-stranded RNA (dsRNA). Exogenous dsRNA triggers sequence-specific degradation of the target endogenous mRNA in the target organisms. dsRNA-mediated RNAi has emerged as one of the most promising tool to study gene function and exhibited tremendous application potential in bio-control of insect pests [2], [3]. So far, several excellent methods have been developed to deliver dsRNA into insects, including microinjection, oral feeding and transgenic expression. In 1998, RNAi mediated by dsRNA injection RNAi was first adopted to investigate gene function in *Drosophila melanogaster* [4]. Because the exact amount of up-take dsRNA can be monitored, so far, microinjection has been extensively used in quite a few insect species such as *Cecropia*, *Acyrthosiphon pisum*, *Nilaparvata lugens*, *Phyllotreta striolata*, etc. In these insects, RNAi have been developed for various genes encoding calreticulin, cathepsin-B, cathepsin-L, hemolin, odorant receptor [2], [5], [6], [7].

Aside from microinjection, artificial feeding is a reliable alternative to deliver dsRNA, especially for small insects, as this method is a non-invasive technique preserving the integrity of the treated animals [6]. In the horticultural pest, *Epiphyas postvittana* (Lepidoptera: Tortricidae), RNAi was triggered by oral delivery of dsRNA to larvae and adult [8]. Ingestion of dsRNA induced RNA interference in several coleopteran species and resulted in larval stunting and mortality [9]. In addition, knockdown of chitin synthase genes in *Anopheles gambiae*, endogenous digestive cellulase enzyme gene and caste-regulatory hexamerin storage protein gene in *Reticulitermes flavipes* has also be realized through dsRNA feeding [10], [11].

Furthermore, gene knockdown by expressing dsRNA in plant has been exploited to control insect pests. For instance, transgenic corn plants expressing western corn rootworm (WCR) *Diabrotica virgifera virgifera vacuolar* ATPase (V-ATPase) subunit dsRNAs showed a significant reduction in WCR feeding damage in a growth chamber assay [9]. When cotton bollworm (*Helicoverpa armigera*) larvae were fed plant material expressing double-stranded RNA (dsRNA) specific to CYP6AE14, a cytochrome P450 gene, levels of its transcript in the midgut decreased and larval growth was retarded [12]. But knockdown of certain genes did not show lethal effect. The hexose transporter gene *NlHT1*, the carboxypeptidase gene *Nlcar* and the trypsin-like serine protease gene *Nltry* are highly expressed in the *Nilaparvata lugens* migut. When *N. lugens* nymphs were fed on rice plants expressing dsRNAs of the three targeted genes, RNA interference was triggered but lethal phenotypic effects after dsRNA feeding were not observed [13].

The gap gene *hb*, which codes for a zinc-finger type transcription factor, is a key regulatory gene in the anteroposterior patterning in a number of insects [14], [15], [16], [17], [18], [19]. *hb* expression can be provided maternally and zygotically. The maternal RNA is distributed homogeneously in the embryo and under the control of the posterior maternal factor *nanos* (*nos*). The zygotic expression of *hb* is under the control of the anterior maternal gene *bicoid* (*bcd*) [20]. In *Drosophila*, loss-of-function alleles for *hb* cause defects in the anterior, including deletions of gnathal and troracic segments [16], [21]. The single depletion of maternal and zygotic *hb* by parental RNAi in both *Tribolium* and *Nasonia* leads to deletion in the head and thorax; knockdown of both *hb* and *orthodenticle* (*otd*), an other gap gene, results in failure to develop the head, trorax and anterior abdomen [18], [22]. In the milkweed bug *Oncopeltus*, the *hb* (*Of*'*hb*) RNAi depletion results in transformations of gnathal and thoracic regions into an abdominal identity, as well as impaired posterior elongation and segmentation [19]. In addition, *hb* is expressed in specific mesodermal cells and in the nervous system. In *Drosophila*, transient *hb* expression can be observed in neuroblasts and in a sub-population of ganglion mother cells (GMCs) and neurons [16], [23]. It is an important determinant in specifying early sublineage identity in the NB7-3 lineage [24].

In this study, we reported that the artificial feeding of *Aphb* dsRNA to the pea aphid depleted the expression of the target gene and decreased insect survival rates. These results suggest that *Aphb* may be a candidate for development of RNAi plants in the control of sap-sucking insects.

## Results

### Sequencing and dsRNA synthesis

**Figure 1:**
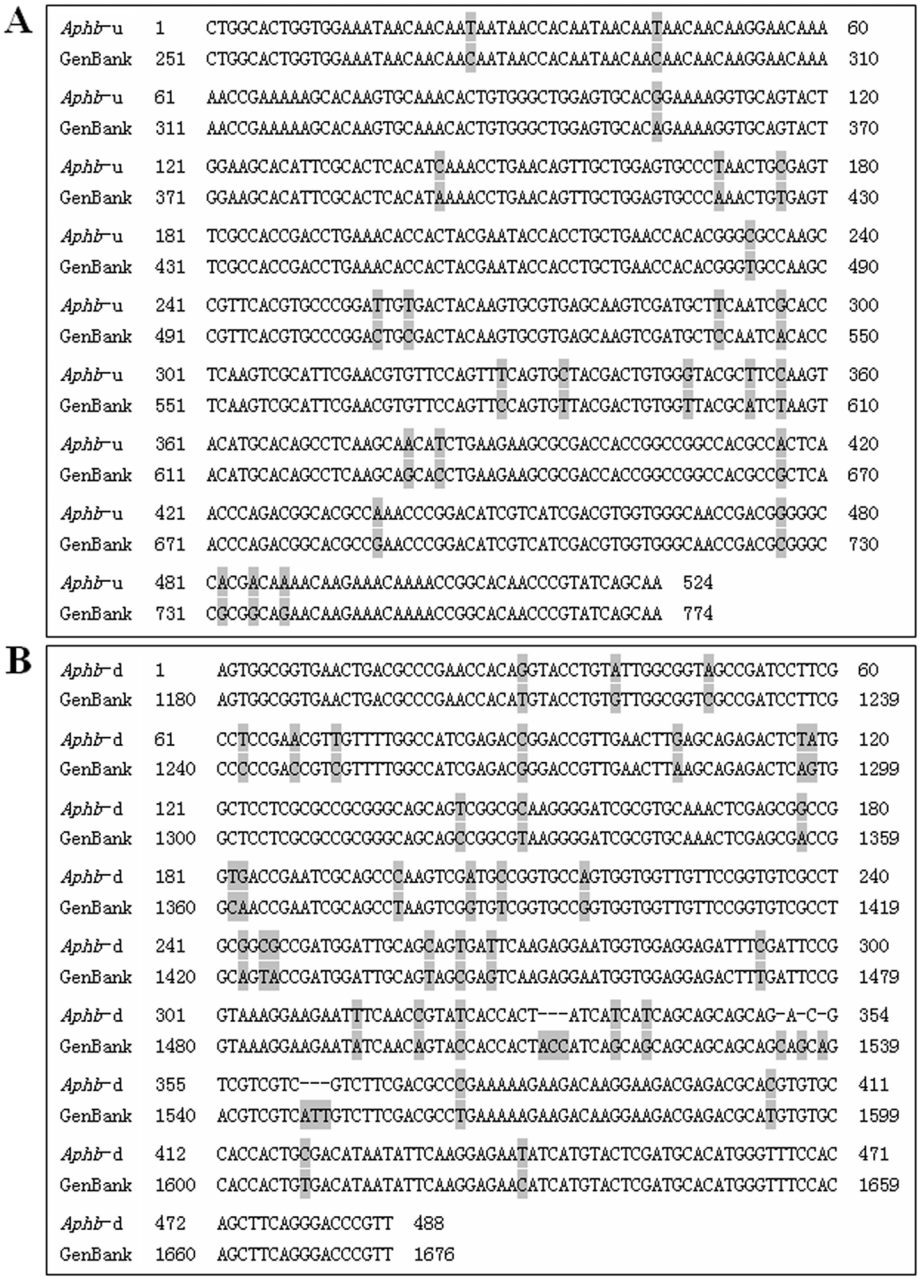
Alignment of the *Aphb* sequence in GenBank. The *Aphb* sequence obtained in present study was run blast in GenBank. (A) *Aphb*-u showed a similarity of 95% with *Acyrthosiphon pisum hb* mRNA (Accession number: NM_001162510.1) in Genbank. (B) *Aphb*-d showed a similarity of 91% with the corresponding sequence of NM_001162510.1. *Aphb*-u, *Aphb* upstream cDNA sequence obtained in present study; *Aphb*-d, *Aphb* downstream cDNA sequence obtained in present study; GenBank, *Aphb* mRNA in GenBank.

The obtained *Aphb*-u target-sequence was 524 bp in size and shows a 95% identity with *Acyrthosiphon pisum hunchback* sequence in GenBank (Accession number NM_001162510.1) (Fig. 1A). The obtained *Aphb*-d target-sequence was 9 bp smaller than the expected 497bp and only showed a 91% identity with NM_001162510.1 (Fig. 1B). So, the PCR products, which composed of *Aphb* fragments and T7 promoter sequence, were 564 bp and 528 bp, respectively, in size. The difference between the obtained sequences in present study and the *Aphb* in GenBank suggested the genetic separation of the pea aphids in different regions. The dsRNA synthesized using MEGAscript® RNAi Kit was purified and quantified spectrophotometrically at 260 nm. Agarose gel electrophoresis revealed that the dsRNAs had good purity and integrity (data not shown).

### *Aphb* expression at different developmental stages

**Figure 2:**
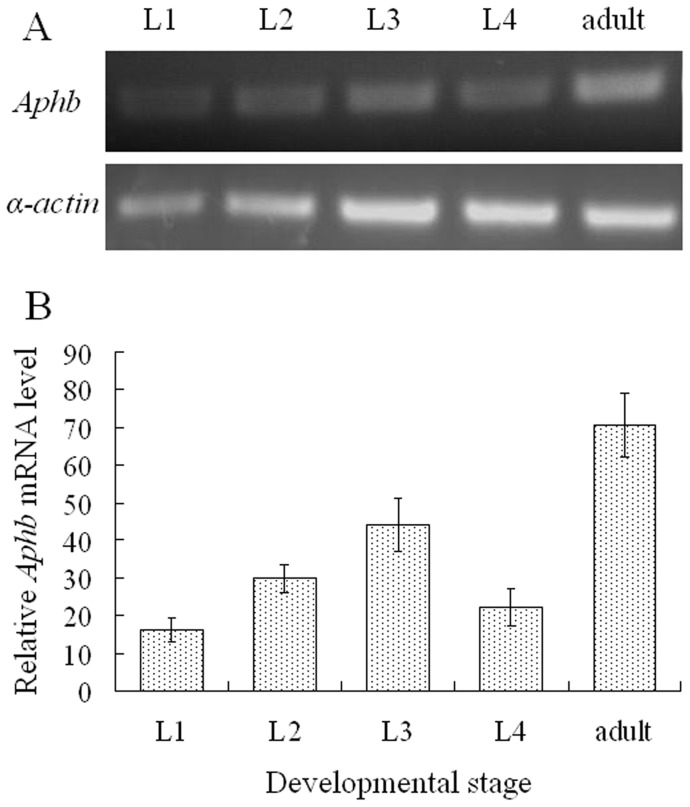
*Aphb* expression in the life cycle of *Acyrthosiphon pisum*. (A) Semiquantitative RT-PCR analysis of the *Aphb* expression. The *Aphb* transcription at different developmental stages was investigated by semi-quantitative PCR. A *α-actin* fragment was amplified for normalization. The *Aphb* expression was most early detected in L1 instar. The mRNA level varied at different stages with a peak occurred at adult stage. (B) Relative expression level of the *Aphb*. Integrated optical density of gene specific bands on agarose gel was analyzed to determinate the *Aphb* transcripts level relative to the *α-actin* expression. The data represent the means ± SE of three replicates.

*Aphb* expression at developmental stages was investigated by semiquantitative RT-PCR. A constitutively expressed *α-actin* gene was used as internal control. Results showed that *Aphb* transcripts accumulated at various levels at different developmental stages (Fig. 2A). From L1 to L3, *Aphb* mRNA level went up with the instar increase. Then the upward trend was interrupted at L4, recovered and peaked at adult stage. Integrated optical density analysis revealed that *Aphb* mRNA levels in L1, L2, L3, L4 and adult relative to the *α-actin* internal control were about 16.2%, 29.8%, 44.1%, 22.2%, 70.6%, respectively (Fig. 2B).

### *Aphb* expression after dsRNA feeding

**Figure 3:**
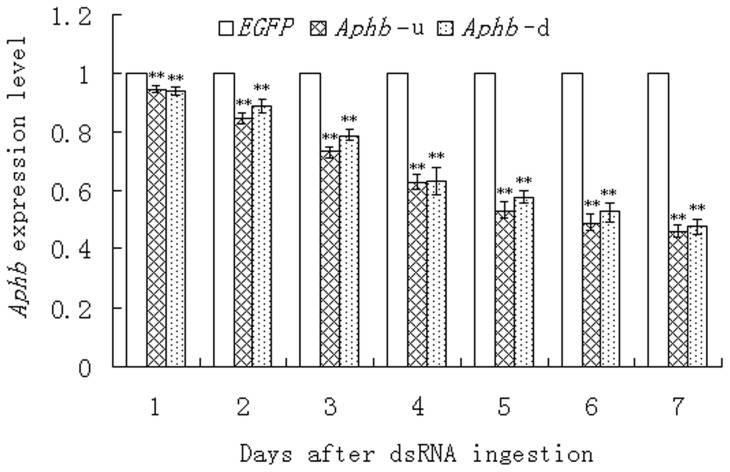
*Aphb* mRNA level after ingestion of dsRNA. *Aph*b transcripts accumulation was analyzed by qRT-PCR over 7 days after dsRNA feeding. The housekeeping gene, *α-actin*, was used as internal control for normalization. Normalised *Aphb* expression was expressed as the proportion of that recorded in the *EGFP* control. Each kinetic point was performed in triplicate on 3 aphids and values are expressed as mean ± SE of three replicates. Double asterisks indicate increasingly significant differences in *Aphb* transcripts levels between the treatment and the control determined by a *t*-test (*p*<0.01).

*Aphb* expression after dsRNA feeding was analyzed by quantitative real-time PCR. At the first day after *Aphb*-u dsRNA feeding, *Aphb* mRNA accumulation showed no obvious reduction. At the third day, ingestion of *Aphb*-u dsRNA resulted in an obvious decrease in *Aphb* mRNA level and the *Aphb* transcripts abundance in *Aphb*-u group was 73% of that in *EGFP* group. Then, the reduction level went up continuously with the elongation of feeding period (Fig. 3) and reached 54% at the seventh day after feeding. During the seven-day feeding assay, the *Aphb* mRNA level showed very significant difference between the *Aphb*-u and *EGFP* groups, but did no show obvious difference between the *Aphb*-u and *Aphb*-d groups.

### Mortality after dsRNA feeding

**Figure 4:**
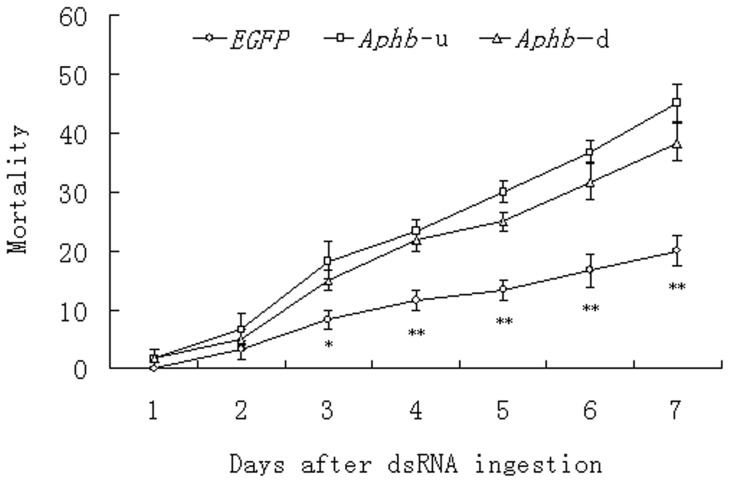
Mortality of pea aphids after dsRNA feeding. L2 nymphs were reared on artificial diet suspended with dsRNA at a final concentration of 0.75 mg/ml. Mortality was recorded at 1 to 7 day after feeding. One asterisk indicates difference of mortality between the treatment and control determined by a *t*-test (*p*<0.05). Double asterisks indicate difference at the *p*<0.01 level. Error bars are standard errors of four independent replicates.

**Figure 5:**
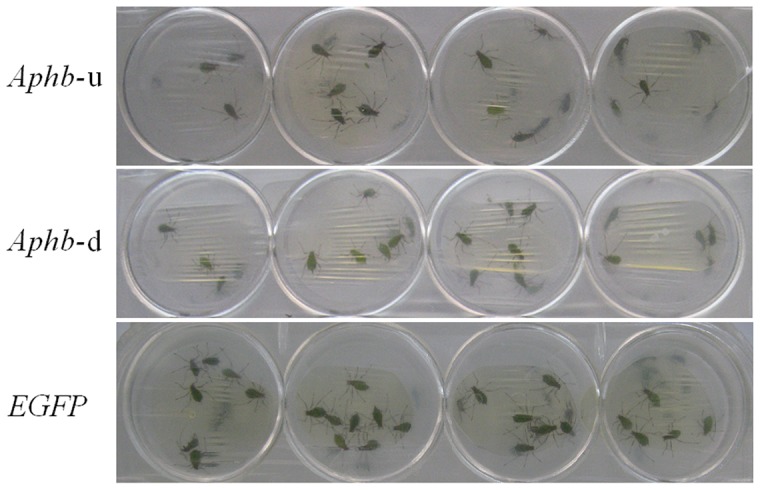
Effect of dsRNA feeding on survival of pea aphids. Small holes were drilled on the bottom of a 24 well culture plate. 15 individuals were transferred into every well of the plate and the openings were sealed with a layer of Parafilm M membrane. The mixture (150 μl) of dsRNA and artificial diet was loaded onto the membrane and then covered with another layer of membrane. On the seventh day after feeding, mortality of the two *Aphb* groups is about 2 times of that of *EGFP* control group. Survival aphids still gathered below the membrane for sucking.

No obvious difference in mortality was observed between *Aphb*-u and the *EGFP* groups on the first and second day post feeding. From the third day onward, the difference became more and more obvious. With the continuous feeding of *Aphb*-u dsRNA, the average mortality reached 18.3%, 23.3%, 30.0%, 36.7% and 45.0% on the third, fourth, fifth, sixth and seventh day, respectively. But in these days, the mortality of *EGFP* group was only 8.3%, 11.7%, 13.3%, 16.7% and 20.0%, respectively. The difference in mortality between the two groups reached significant level on the third day and became increasingly significant from the fourth day to the seventh day after feeding. The *Aphb*-u group showed a slightly higher mortality than the *Aphb*-d group, but the difference between them did not reach significant level during the whole dsRNA feeding period (Fig. 4). On the seventh day after dsRNA feeding, more control insects survived and gathered below the membrane for sucking when compared with silenced groups (Fig. 5). These results suggested that the enhanced insect mortality was resulted from continuous feeding of *Aphb* dsRNA.

## Discussion

In this paper, we investigated the *Aphb* expression level at different developmental stages of the pea aphid, *Acyrthosiphon pisum*. The *Aphb* transcripts were most early detected at the first instar stage. We also conducted RNAi by dsRNA ingestion to deplete the *Aphb* expression in the adult sap-sucking insect. A reduction of *Aphb* mRNA accumulation and an increase of insect mortality were observed in the dsRNA feeding assay. Our finding showed that the *Aphb* knockdown mediated by dsRNA ingestion was lethal to the pea aphid.

This work is the first report on RNAi mediated by dsRNA ingestion in pea aphid. Though *Aphb* transcripts were detected at L1 stages, L2, not L1 instar nymphs were tested in dsRNA feeding assay. This is because mortality is very high when new-born nymphs were fed on artificial diet directly without a pre-rearing step in petri dish. The RNAi efficiency is relatively high in present study when compared with RNAi mediated by microinjection. When each pea aphid of L3 instar was injected with 0.276 μg dsRNA on the third instar stage, a maximum reduction of 41% of the target gene was achieved at 5 days post injection [2]. In present study, the *Aphb* expression level dropped 54% at 7 days after dsRNA ingestion. RNAi efficiency may vary on different target genes. Another explanation may be that dsRNA transported by oral feeding is more efficient in pea aphid than that delivered by microinjection. In order to test the effect of different target-sequences, both a conserved upstream segment (*Aphb*-u) and a downstream sequence (*Aphb*-d) of the *Aphb* were used as templates for dsRNA synthesis in the study. We found that the *Aphb*-u dsRNA resulted in slightly higher rate of mortality than the *Aphb*-d dsRNA did. But the difference was not statistically significant, suggesting that the targeted region is not the decisive factor affecting the RNAi efficiency in *Aphb* silence.

The *hb* gene, which has been found in various species of insects, is an important player in the early embryonic anteroposterior patterning [15], [16], [17], [18], [19], [20], [25], [26]. Depletion of this function resulted in abnormity and death of embryos. In addition, *hb* plays important roles in sequential cell fate specification within the *Drosophila* central nervous system (CNS). Its expression was found in ventral nerve cords of L1 larval stage [24]. *Drosophila* is a model insect. Due to the accessibility and relative simplicity, *Drosophila* ventral nerve cord (VNC) is a good model system to elucidate the roles of *hb* in CNS development. Up to now, almost nothing is known about how the *hb* functions in CNS development of other insects. A question we have to answer here was which function of the *Aphb* was depleted in present RNAi experiment performed at L2 instar stage. Patterning the embryonic segmentation or specifying the development of central nervous system, or both of them? It is difficult to relate the lethal effect to *Aphb* roles in patterning the embryogenesis. Though the function in specifying the CNS development has not been confirmed present in *A. pisum*, it is likely the reason why the insect survival rate decreased in the ingested RNA interference. Further study is needed to confirm this hypothesis.

RNA interference (RNAi) is a mechanism for post-transcriptional gene silencing and has shown us attractive prospect in management of agricultural pests. According to reports, *cathepsin*-B gene [6], *actin* gene [27], [28], *trehalose phosphate synthase* gene (TPS) [29] and odorant receptor gene (Or) [7] are potential RNAi targets. Each of these genes plays a single function in insects and knockdown of them always resulted in a certain phenotype. But the gap gene *hb* is different in this aspect as it has dual functions. Its roles in anteroposterior patterning have been much reported in a number of insects and its functions in specifying the CNS development might exist in other insects aside from *Drosophila*. If these two different functions were disrupted, single knockdown of the *hb* gene should show dual silencing effects and result in higher mortality. In addition, depletion of the CNS development is aimed at dsRNA treated generation, but disruption of embryogenetic segmentation is designed to suppress the offspring. So, silencing of *hb* is able to affect both the present generation and the progenies. In a word, the *hb* gene is a very excellent candidate of RNAi target in management of insect pests.

So far, RNA interference (RNAi) has been applied extensively to study gene function in a variety of organisms. dsRNA can be delivered to insects through oral feeding, microinjection, soaking and transgenic expression [6], [9], [12], [30], [31], [32], [33]. Microinjection is a widely used method and able to realize RNAi with high efficiency in a number of insects, including pea aphid [2], [6], [18], [19], [25]. But this method is often accompanied with high mortality especially in small insects. Furthermore, the penetrance of dsRNA delivered by injection is not high in certain insect species [19]. This work is to investigate whether the *hb* gene can be used as a RNAi target in the control of pea aphid. In natural conditions, dsRNA is much easier to be transported into insects by oral feeding than by physical contact and infiltration. Considering this, oral feeding was adopted to realize RNAi in present study. A problem we encountered is that artificial diet is not as nutritious as fresh plant seedlings and aphids reared on it developed slowly and laid few nymphs. Thus, the effect of *Aphb* depletion on embryonic segmentation can not be analyzed simultaneously. The best solution to this problem is to construct transgenic plants expressing *Aphb* dsRNA. In this way, the *hb* functions both in patterning the embryonic segmentation and specifying the CNS development are expected to be depleted in a single experiment. Furthermore, this kind of engineering plants is likely to suppress both CNS development and embryogenesis of insect pests. Another problem we have to mention is that the similarity between the *Aphb* sequences in present study and that in GenBank is unexpectedly not very high. This phenomenon is a commonplace and can be explained by genetic isolation because insect samples used for gene cloning are from different geographical regions. RNAi is a sequence specific silencing mechanism of great accuracy. High interference efficiency can be achieved only when the designed dsRNA sequence is highly identical with the target-sequence in organism. In our preliminary experiment, dsRNA used for artificial feeding was designed directly by using *Aphb* mRNA sequence in GenBank as template. Consequently, the RNAi efficiency was low after feeding (results not shown). When the *Aphb* dsRNA was synthesized based on our sequencing results, high interference efficiency was obtained. So, genetic difference resulted from geographic isolation should be taken into consideration in sequence specific RNAi application.

Oogenesis and embryogenesis of insects are under the control of a series of genes, including *hunchback*, *orthodenticle* (*otd*), *bicoid* (*bcd*), *oskar* (*osk*), etc. These genes play indispensable roles and single knockdown of them resulted in defects and even death in the next generation [18], [19], [34], [35]. Theoretically, all these genes are good RNAi candidates in the fight against insect pests.

## Materials and Methods

### Experimental insects

The colony of *Acyrthosiphon pisum* used in the study was a laboratory strain kindly provided by Dr. Zhang Fan, Beijing Academy of Agriculture and Forestry Sciences. Insects were reared on broad bean plants at 26–27°C under a 16∶8 h light:dark photoperiod. New broad bean seedlings were provided once a week.

### *Aphb* expression analysis

Semiquantitative RT-PCR was performed to analyze the *Aphb* expression level at the first, second, third, fourth instar (L1, L2, L3, L4) stage and adult stage. Total RNAs were isolated from 60 L1, 30 L2, 20 L3, 10 L4 instars or 5 adults using Tranzol reagents (Transgene, Beijing, China). DNA contaminations were removed by digesting RNA solution with DNase (Ambion, Texas, USA). cDNA was synthesized using TransScript First-Strand cDNA Synthesis SuperMix (Transgene, Beijing, China) with anchored Oligo(dT)_18_ primer. Gene specific primers were designed to amplify a 240 bp of the *Aphb* mRNA. A *α-actin* (GenBank Accession Number: XM_001950723.2), a constitutively expressed gene, was used as internal control. Primers used in semiquantitative RT-PCR are shown in Table 1.

**Table 1:** Primers used in the experiments.

Gene or fragment	PCR type	Forward	Reverse	Product size (bp)
*Aphb*-u	RT-PCR^a^	TAATACGACTCACTATAGGG CTGGCACTGGTGGAAATA	TAATACGACTCACTATAGGG TTGCTGATACGGGTTGTG	564
*Aphb*-d	RT-PCR^a^	TAATACGACTCACTATAGGG AGTGGCGGTGAACTGACG	TAATACGACTCACTATAGGGAACGGGTCCCTGAAGCT-3′	528
*EGFP*	RT-PCR^a^	TAATACGACTCACTATAGGG CCACAAGTTCAGCGTGTCCG	TAATACGACTCACTATAGGG AAGTTCACCTTGATGCCGTTC	463
*Aphb*	Semi- quantitative PCR^b^	CTGGCACTGGTGGAAATAA	TGTGGTTCAGCAGGTGGTAT	228
*α-actin*	Real-time PCRb, c	CAATGGGACAGATTAGGTAG	AGCATCCGACAAAGTAGC	240
*Aphb*	Real-time PCR^c^	AAGCACATTCGCACTCACA	GTTCAGCAGGTGGTATTCGT	102

a Primers used in dsRNA synthesis for amplification of the target fragments.

b Primers used in Semi-quantitative PCR for mRNA level detection.

c Primers used in qRT-PCR for mRNA level detection.

For each gene, PCR products in separate tubes were analyzed after 24, 25, 26, 27, 28, 29, 30, 32, 34 and 36 cycles by gel electrophoresis. The threshold cycle was determined as the cycle at which visible band of specific PCR product first appeared on the ge1. The amplifications of *Aphb* and *α-actin* were performed with 35 cycles. Integrated optical density of gene specific bands on agarose gel was analyzed by GelPro4.0 (Media Cybernetics, MD, USA) to determinate the *Aphb* transcripts level at different stages. The experiment was repeated three times and all values were the means of three individual measurements ± SE.

### *Aphb* dsRNA synthesis

The dsRNAs were synthesized in vitro using MEGAscript® RNAi Kit (Ambion, Texas, USA). Two *Aphb* coding fragments were selected as RNAi target-sequences. The upstream one (*Aphb*-u) was 524 bp (251–774 bp) containing the conserved motifs MF1-4 and C-box sequence. The downstream one (*Aphb*-d) was 497 bp (1180–1676). T7 primers (T7 promoter plus exon specific sequence) were designed according to the Instruction Manual of the MEGAscript® RNAi Kit. The PCR of these two target fragments was performed at 94°C for 3 min, followed by 35 cycles of 94°C for 30 s, 56.2°C for 30 s, and 72°C for 40 s, finishing with an extension step at 72°C for 10 min. PCR products were purified using TIANgel Midi Purification Kit (Tiangen, Beijing, China) and sequenced. dsRNA was synthesized by using the PCR product as template and then purified with DNase/RNase digestion. The purified dsRNA was quantified spectrophotometrically at 260 nm, subjected to agarose gel electrophoresis to determine purity and integrity and stored at −80°C before use. *EGFP* (GenBank Accession Number: CVU55761) dsRNA was also synthesized as above procedures with *EGFP* specific primers (T7 promoter plus *EGFP* specific sequence) and severed as a control in artificial feeding. Primers used in the dsRNA synthesis for amplification of the target gene are shown in Table 1.

### Artificial feeding of dsRNA

2 day old nymphs were fed on dsRNA-contained diet and mortality was recorded daily. The diet used for aphid rearing was a meridic artificial diet [36] with a sucrose content lowered to 20% [37]. 24 well culture plates (Sigma, Germany) were used as rearing device. At first, small holes were made on the bottom of the culture plate for ventilation. Neonate nymphs were pre-reared on detached broad bean leaves for two days in petri dish. 15 individuals were transferred into every well of the plate using a writing brush and the plate was sealed with stretched Parafilm M membrane (Pechiney Plastic Packaging Company, Chicago, USA). The dsRNA was incorporated to the artificial diet as a supplement and its final concentration was designated as 0.75 mg/ml. The mixture of dsRNA and artificial diet was loaded onto the stretched membrane above wells and then covered with another layer of stretched Parafilm membrane. Aphids could puncture the inner layer of Parafilm M membrane and feed on the mixture dispensed between the two layers of membrane. Then the plate was covered and located in greenhouse kept at 26–27°C under a light:dark regimen of 16∶8 h. The plate and mixture were renewed every day. Mortality rate of the pea aphids was checked daily for statistics (1–6 days). The artificial feeding bioassay was repeated for three times.

### *Aphb* silencing analysis

The accumulation of *Aphb* mRNA after dsRNA feeding was investigated by qRT-PCR using an IQ-5 Real-Time System (Bio-Rad, California, USA). Total RNAs were isolated from feeding aphids and cDNA was synthesized according to above procedures. qRT-PCR was performed using a final volume of 25 μl containing cDNA produced from 2 μg total RNA, 11.25 μl of SYBR® Green Real-time PCR Master Mix (TOYOBO, Japan) and 200 nM each of forward and reverse *Aphb* specific primers. Primers used in the qRT-PCR for mRNA level detection are shown in Table 1.

qRT-PCR was performed under following program: one cycle of 95°C for 60 s; then 40 cycles of 95°C for 15 s, 51.5°C for 15 s and 72°C for 45 s. Standard curves were obtained using a 10-fold serial dilution of the cDNAs pooled from 5 adults reared on broad bean seedling. Three technical replicates of each reaction were performed and *α-actin* (GenBank Accession Number: XM_001950723.2), a constitutively expressed gene, was used as internal control for normalization. Means and standard errors for each time point were obtained from the average of three independent sample sets. Quantification of the relative changes in gene transcript level was performed according to the 2^−ΔΔCt^ method [38].
